# Factors associated with gay-affirmative practice among polish paramedics: a cross-sectional study

**DOI:** 10.3389/fpubh.2026.1919205

**Published:** 2026-09-03

**Authors:** Michał Czapla, Piotr Karniej, Raúl Juárez-Vela, Krzysztof Griesmann, Kamil Kędzierski, Aleksander Mickiewicz, Antonio Martínez-Sabater, Anthony Dissen, Łukasz Pietrzykowski, Jacek Smereka

**Affiliations:** 1 Department of Emergency Medical Service, Faculty of Nursing and Midwifery, Wroclaw Medical University, Wrocław, Poland; 2 Group for Research in Healthcare (GRUPAC), Faculty of Health Science, University of La Rioja, Logroño, Spain; 3 Faculty of Nursing and Podology, Nursing Care and Education Research Group (GRIECE), University of Valencia, València, Spain; 4 Faculty of Finance and Management, WSB MERITO University in Wrocław, Wrocław, Poland; 5 Care Research Group (INCLIVA), Hospital Clínico Universitario de Valencia, València, Spain; 6 School of Health Sciences, Stockton University, Galloway, NJ, United States; 7 Department of Anaesthesiology and Intensive Care, Antoni Jurasz University Hospital No. 1 in Bydgoszcz, Bydgoszcz, Poland

**Keywords:** emergency medical services, gay-affirmative practice, health equity, LGBT patients, paramedics

## Abstract

**Introduction:**

Gay-affirmative practice is considered an important component of equitable healthcare for sexual minority patients. However, little is known about the factors associated with affirmative beliefs and professional behaviors among paramedics. This study aimed to identify factors associated with gay-affirmative beliefs and behaviors among Polish paramedics, with particular emphasis on the role of LGBT clinical competence.

**Methodology:**

A cross-sectional online survey was conducted between January and June 2025 among practicing paramedics in Poland. Participants completed a survey comprised of a sociodemographic questionnaire, the Polish version of the Lesbian, Gay, Bisexual, and Transgender Development of Clinical Skills Scale (LGBT-DOCSS), and the Polish version of the Gay Affirmative Practice Scale (GAP). Univariable and multivariable linear regression analyses were performed to identify factors independently associated with the Beliefs and Behaviors domains of the GAP. Clinical trial number: not applicable.

**Results:**

A total of 172 paramedics were included in the final analysis (mean age 29.4 years, 51.2% women). Higher LGBT clinical competence was independently associated with both more affirmative beliefs (*β* = 3.85, 95% CI: 1.21–6.50, *p* = 0.005) and behaviors (*β* = 7.29, 95% CI: 4.62–9.96, *p* < 0.001). Having close relationships with LGBT individuals was independently associated with more affirmative beliefs (*β* = 6.40, 95% CI: 1.97–10.84, *p* = 0.005), whereas right-wing political orientation was independently associated with lower affirmative beliefs (*β* = −12.26, 95% CI: −17.53 to −6.99, *p* < 0.001) and behaviors (*β* = −6.74, 95% CI: −12.07 to −1.41, *p* = 0.013).

**Conclusion:**

LGBT clinical competence was the factor most consistently associated with gay-affirmative beliefs and behaviors among Polish paramedics. These findings suggest that higher LGBT clinical competence may represent an important factor associated with more affirmative beliefs and behaviors. Future studies should evaluate whether educational interventions aimed at strengthening LGBT clinical competence translate into more affirmative prehospital care for LGBT patients.

## 1 Introduction

Lesbian, gay, bisexual, transgender and other sexual and gender minority (LGBT+) individuals experience persistent health inequalities relative to their heterosexual and cisgender counterparts, including poorer mental and physical health outcomes, higher rates of substance use, and an elevated risk of self-harm and suicide (1, 2). These disparities are largely driven by minority stress, stigma, and discrimination, and are further compounded by heteronormativity within healthcare systems (1, 3). LGBT+ individuals encounter structural and interpersonal barriers when accessing care, including heteronormative assumptions, fear of disclosure, and discriminatory treatment by healthcare professionals (4–6). As a result, they are considerably more likely than heterosexual individuals to delay or avoid care and to report unmet healthcare needs, frequently because of previous negative encounters with providers (7–9). This avoidance contributes to delayed diagnoses, poorer health outcomes, and reduced trust in the healthcare system (9, 10). Importantly, the quality of care that LGBT+ individuals receive depends not only on legal protections and institutional policies, but also on the knowledge, attitudes, and behaviors of healthcare professionals-provider-level factors that have emerged as potentially modifiable determinants of equitable and inclusive care (3, 5, 11).

In Poland, LGBT+ individuals continue to face an unfavorable legal and social environment (12). In the 2026 edition of ILGA-Europe’s Rainbow Map, which ranks 49 European countries according to their LGBT+-related laws and policies, Poland scored 21.5% and ranked among the lowest-performing European Union member states, whereas Spain was ranked first (88.7%) (13). However, such indicators primarily reflect legal and policy frameworks rather than the quality of healthcare delivered in everyday clinical practice, which is influenced by the competencies of individual healthcare professionals. Consistent with this distinction, two recent cross-national studies found that healthcare professionals in Spain reported significantly higher LGBT clinical competence (14) and more affirmative beliefs and behaviors toward gay and lesbian patients (15) than their Polish counterparts, while profession, gender, age, and participation in LGBT-related training were independently associated with these outcomes. These findings suggest that national legal contexts may not fully explain variations in healthcare experiences, and underscore the importance of examining individual-level factors that influence healthcare delivery to sexual and gender minority populations.

LGBT clinical competence has emerged as an important framework for understanding healthcare professionals’ preparedness to provide equitable and inclusive care for LGBT patients. This construct encompasses knowledge, attitudes, and clinical preparedness related to the health needs of LGBT populations and has been increasingly assessed using the LGBT Development of Clinical Skills Scale (LGBT-DOCSS) (16). Previous studies have shown that healthcare professionals and students often report limited formal education in LGBT health and varying levels of competence in addressing the needs of LGBT individuals, potentially contributing to disparities in healthcare experiences and outcomes (17–20). Beyond clinical competence, the concept of gay-affirmative practice reflects the extent to which healthcare professionals endorse and implement affirmative beliefs and behaviors toward gay and lesbian patients (15, 21, 22). Although both constructs are considered important for the delivery of patient-centered and equitable care, relatively little is known about the relationship between LGBT clinical competence and gay-affirmative practice among healthcare professionals in Central and Eastern European settings, including Poland.

This knowledge gap may be particularly relevant in prehospital care, where paramedics frequently provide care in time-critical, unpredictable, and highly sensitive situations, often representing patients’ first and sometimes only point of contact with the healthcare system (17, 23, 24). Our previous national study of Polish paramedics found that, despite relatively positive attitudes toward LGBT patients, clinical preparedness was limited and was associated with factors such as recent LGBT-related training, prior informal contact with LGBT individuals, and sex (17). However, that study focused exclusively on LGBT clinical competence, and whether such competencies translate into affirmative beliefs and behaviors toward gay and lesbian patients remains unknown. Given the central role of paramedics in emergency care, understanding the factors associated with gay-affirmative practice in this professional group may help identify opportunities to improve equitable healthcare delivery for gay and lesbian patients.

The aim of this study was to identify factors associated with gay-affirmative beliefs and behaviors among Polish paramedics, with particular emphasis on the role of LGBT clinical competence.

## 2 Methodology

### 2.1 Study population

This cross-sectional study was conducted among practicing paramedics in Poland between January and June 2025. Data were collected using an anonymous online survey administered through the Webankieta.pl. platform. To minimize the risk of duplicate responses and enhance data quality, the survey platform incorporated IP address verification, preventing multiple submissions from the same device.

Before accessing the questionnaire, all participants received detailed information regarding the study objectives, voluntary nature of participation, confidentiality of the collected data, and their right to withdraw at any stage without providing a reason. Electronic informed consent was obtained from all participants prior to survey completion.

Eligible participants were certified paramedics aged 18 years or older who were actively practicing in Poland at the time of the study. Participants were recruited using a non-probability convenience sampling strategy. The survey link was distributed through professional and social media channels, including Facebook and Instagram, and further disseminated through professional networks of the research team.

**Figure 1:**
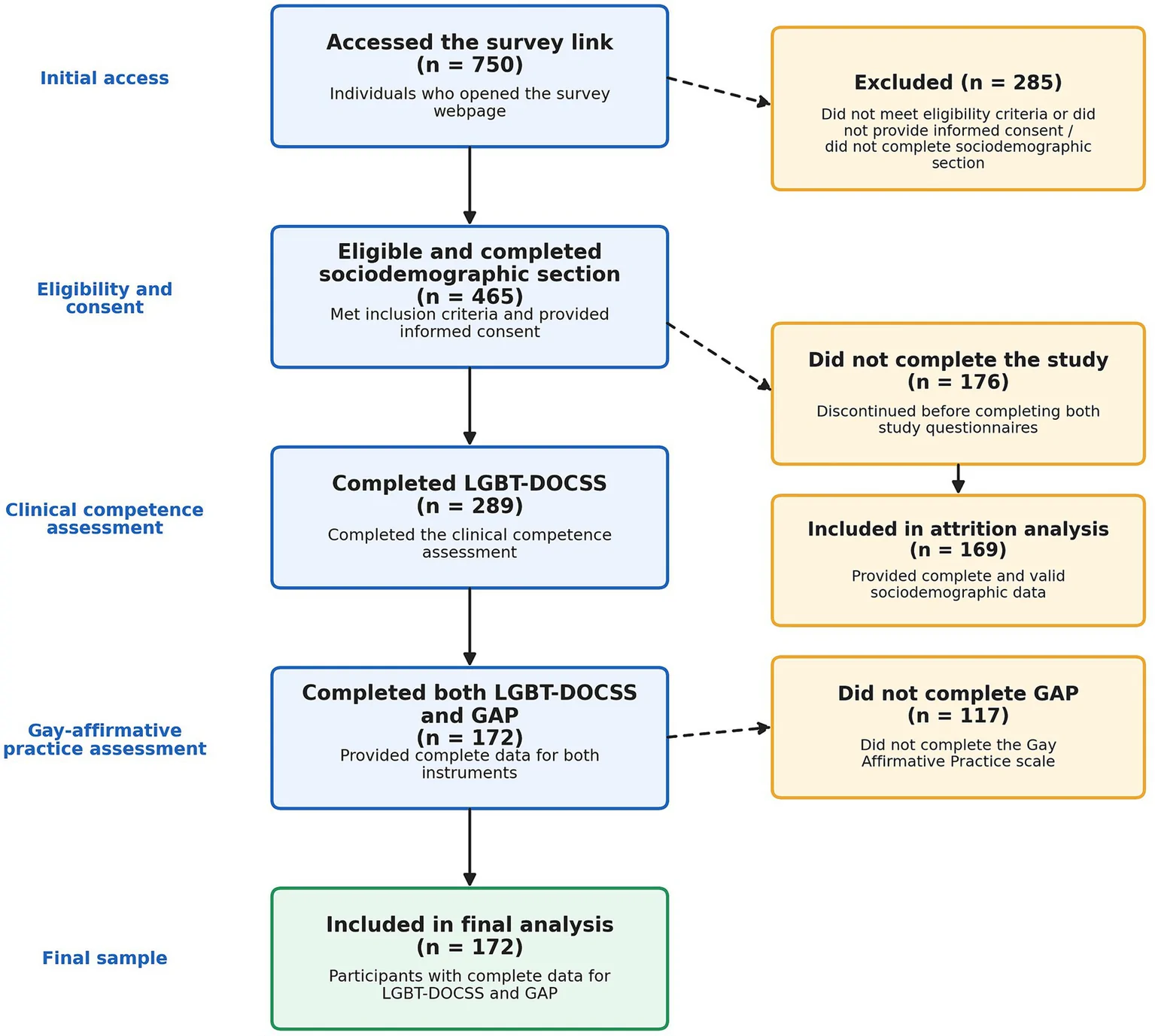
Flow diagram of participant selection. LGBT-DOCSS, LGBT Development of Clinical Skills Scale; GAP, Gay Affirmative Practice.

Overall, 750 individuals accessed the survey webpage. Among them, 465 participants met the eligibility criteria and completed the informed consent and sociodemographic sections of the questionnaire. Subsequently, 289 respondents completed the LGBT Development of Clinical Skills Scale (LGBT-DOCSS). Of these, 172 participants additionally completed the Gay Affirmative Practice (GAP) scale. Because the primary objective was to investigate factors associated with gay-affirmative beliefs and behaviors while accounting for LGBT clinical competence, only participants who completed both the LGBT-DOCSS and the GAP were included in the final analytical sample (n = 172). The participant selection process is presented in Figure 1. This study was reported in accordance with the Strengthening the Reporting of Observational Studies in Epidemiology (STROBE) statement.

### 2.2 Measures

A structured sociodemographic questionnaire developed for this study was used to collect participant characteristics. The questionnaire included information on gender, age, place of residence, principal place of work, sexual orientation, marital status, education level, professional experience, average monthly working hours, previous exposure to LGBT health topics during formal education, participation in LGBT-related training activities, experience working with LGBT patients, and self-perceived preparedness to provide care for LGBT individuals.

LGBT clinical competence was assessed using the Polish version of the LGBT-DOCSS, adapted and validated by Karniej et al. (16, 25). The instrument consists of 18 items rated on a 7-point Likert scale and evaluates healthcare professionals’ development of competencies related to LGBT patient care. The scale comprises three domains: Clinical Preparedness, Attitudes, and Knowledge. Following the scoring procedure recommended by the original authors, negatively worded items were reverse-coded before analysis. Higher scores indicate greater preparedness for providing care to LGBT patients, more favorable attitudes toward LGBT individuals, and higher levels of LGBT-related clinical knowledge. In the validation study of the Polish version, the instrument demonstrated satisfactory psychometric properties, with a Cronbach’s alpha coefficient of 0.789 for the overall scale and McDonald’s omega of 0.86 (25).

The Polish version of the Gay Affirmative Practice scale (GAP-PL) was used to assess affirmative beliefs and professional behaviors related to the provision of care for sexual minority individuals. The Polish version of the instrument, adapted and validated by Karniej et al (21, 26), consists of two domains evaluating affirmative beliefs and affirmative behaviors in professional practice. Higher scores indicate greater endorsement of affirmative attitudes and more frequent implementation of affirmative practices. The Polish adaptation demonstrated excellent psychometric properties, with Cronbach’s alpha coefficients ranging from 0.936 to 0.949 and a McDonald’s omega coefficient of 0.963 (26). Although LGBT-DOCSS assesses broader competencies related to the care of LGBT patients, the GAP instrument specifically evaluates affirmative beliefs and behaviors toward gay and lesbian patients. Accordingly, associations involving gay-affirmative practice should be interpreted within this narrower conceptual scope.

### 2.3 Statistical analyses

Continuous variables were summarized using means and standard deviations (SD), as well as medians, interquartile ranges (IQR), minimum values, and maximum values, as appropriate. Categorical variables were presented as absolute frequencies and percentages. To assess potential attrition bias, baseline characteristics were compared between participants included in the final analytical sample and participants who did not complete the study but provided complete and valid sociodemographic data. Categorical variables were compared using the χ^2^ test or Fisher’s exact test, whereas continuous variables were compared using the Mann–Whitney U test. To identify factors associated with gay-affirmative beliefs and behaviors, univariable and multivariable linear regression analyses were performed. Regression coefficients (*β*) with corresponding 95% confidence intervals (95% CI) were reported. Variables demonstrating statistically significant associations in univariable analyses (*p* < 0.05) were considered for inclusion in multivariable models. Because the primary objective of this study was to evaluate the association between overall LGBT clinical competence and gay-affirmative practice, only the overall LGBT-DOCSS score was considered for inclusion in the multivariable models, whereas its individual domains (Clinical Preparedness, Attitudes, and Knowledge) were examined only in the univariable analyses. Separate multivariable linear regression models were constructed for the GAP Beliefs and GAP Behaviors domains. Multicollinearity among independent variables was assessed using the variance inflation factor (VIF). Variables with VIF values greater than 5 were considered collinear and excluded from the final multivariable models. A two-sided *p*-value < 0.05 was considered statistically significant. All analyses were conducted using R statistical software (version 4.6.0; R Foundation for Statistical Computing, Vienna, Austria).

## 3 Results

### 3.1 Participant characteristics

The final analytical sample comprised 172 paramedics (mean age 29.44 ± 5.86 years), of whom 51.16% were women. Of the 176 participants who did not complete the study, 169 provided complete and valid sociodemographic data and were therefore included in the attrition analysis. Compared with participants who did not complete the study, those included in the final analytical sample were more likely to report a non-heterosexual orientation, previous experience working with LGBT patients outside formal training, greater self-perceived preparedness to work with LGBT patients, close relationships with LGBT individuals, non-religious affiliation, more left-leaning political views, and a greater belief that personal beliefs may influence professional practice (all *p* < 0.05). No statistically significant differences were observed between groups with respect to age, gender, place of residence, principal place of work, marital status, education, work experience, average monthly working hours, or previous LGBT-related education and training (all *p* > 0.05). Detailed characteristics of both groups are presented in Table 1.

**Table 1:** Baseline characteristics of participants included in the final analytical sample and participants who did not complete the study.

Parameter		Final sample (*N* = 172)	Incomplete responses (*N* = 169)**	*p*
Gender	Female	88 (51.16%)	70 (41.42%)	0.065
	Male	83 (48.26%)	99 (58.58%)	
	Other	1 (0.58%)	0 (0.00%)	
Age [years]	Mean (SD)	29.44 (5.86)	28.54 (4.78)	0.429
	Median (quartiles)	27.5 (25–32.25)	27 (25–31)	
	Range	23–50	22–46	
	*n*	172	169	
Place of residence	Rural area	23 (13.37%)	30 (17.75%)	0.126
	City up to 20 th. inhab.	8 (4.65%)	14 (8.28%)	
	City 20–100 th. inhab.	31 (18.02%)	31 (18.34%)	
	City 100–500 th. inhab.	32 (18.60%)	39 (23.08%)	
	City over 500 th. inhab.	78 (45.35%)	55 (32.54%)	
Principal place of work	Emergency Medical Team	96 (55.81%)	90 (53.25%)	0.872
	Emergency Department	48 (27.91%)	54 (31.95%)	
	Hospital ward other than the ED	13 (7.56%)	12 (7.10%)	
	Other	15 (8.72%)	13 (7.69%)	
Sexual Orientation	Heterosexual	126 (73.26%)	155 (91.72%)	<0.001*
	Homosexual	21 (12.21%)	9 (5.33%)	
	Bisexual	23 (13.37%)	3 (1.78%)	
	I’d rather not to tell/other	2 (1.16%)	2 (1.18%)	
Marital status	Single, divorced	48 (27.91%)	60 (35.50%)	0.275
	Married or in formalized partnership	49 (28.49%)	47 (27.81%)	
	Informal relationship	75 (43.60%)	62 (36.69%)	
Education	High school or bachelor’s degree	125 (72.67%)	127 (75.15%)	0.692
	Master’s degree or higher	47 (27.33%)	42 (24.85%)	
Work experience [years]	Mean (SD)	5.68 (4.72)	4.6 (3.59)	0.065
	Median (quartiles)	4 (2–7)	4 (2–5)	
	Range	0–21	1–20	
	n	172	169	
Average number of working hours per month [h]	Mean (SD)	212.94 (55.45)	209.66 (63.73)	0.601
	Median (quartiles)	200 (170–240)	200 (168–250)	
	Range	96–360	72–400	
	n	172	169	
Has the topic of LGBT health been addressed in your previous education (studies/post-secondary education)?	No,	137 (79.65%)	132 (78.11%)	0.773
	Yes	26 (15.12%)	25 (14.79%)	
	I do not remember	9 (5.23%)	12 (7.10%)	
LGBT-related trainings (conferences, webinars) in last 5 years	No	165 (95.93%)	162 (95.86%)	1
	Yes	7 (4.07%)	7 (4.14%)	
Experience working with LGBT patients outside of formal training	No	44 (25.58%)	82 (48.52%)	<0.001*
	Yes	128 (74.42%)	87 (51.48%)	
Do you feel prepared to work with LGBT patients in terms of content and practice?	No	43 (25.00%)	67 (39.64%)	0.005*
	Yes	129 (75.00%)	102 (60.36%)	
Religious beliefs	Non-religious	77 (44.77%)	43 (25.44%)	<0.001*
	Religious	95 (55.23%)	126 (74.56%)	
Close relationships with LGBT persons (friends, family)	No	41 (23.84%)	81 (47.93%)	<0.001*
	Yes	131 (76.16%)	88 (52.07%)	
Overall political views	Centrist	53 (30.81%)	32 (18.93%)	0.001*
	Left-wing	44 (25.58%)	25 (14.79%)	
	Right-wing	30 (17.44%)	44 (26.04%)	
	I cannot assess	45 (26.16%)	68 (40.24%)	
Do you think your personal beliefs can influence the way you work with LGBT people?	No	92 (53.49%)	115 (68.05%)	0.008*
	Yes	80 (46.51%)	54 (31.95%)	

*p* values were calculated using the *χ*^2^ test or Fisher’s exact test for categorical variables and the Mann–Whitney U test for continuous variables. *Statistically significant (*p* < 0.05). **Of the 176 participants who did not complete the study, 169 provided complete and valid sociodemographic data and were included in the attrition analysis. Seven participants were excluded because of incomplete or invalid sociodemographic responses.

### 3.2 LGBT-DOCSS and GAP-PL results

The mean overall LGBT-DOCSS score was 4.51 (SD = 0.87). Among the LGBT-DOCSS domains, the highest mean score was observed for Attitudes (5.73 ± 1.44), followed by Knowledge (4.13 ± 1.51), whereas the lowest score was recorded for Clinical Preparedness (3.50 ± 1.24). For the GAP-PL scale, the mean score for the Beliefs domain was 56.33 (SD = 14.17), while the mean score for the Behaviors domain was 48.56 (SD = 14.45). Detailed descriptive statistics for the LGBT-DOCSS and GAP scales are presented in Table 2.

**Table 2:** Descriptive statistics for LGBT-DOCSS and GAP scale scores among the study participants.

LGBT-DOCSS	*N*	Mean	SD	Median	Min	Max	Q1	Q3
Total LGBT-DOCSS score	172	4.51	0.87	4.44	2.11	6.78	3.89	5.11
Clinical preparedness	172	3.50	1.24	3.57	1.14	6.57	2.57	4.14
Attitudes	172	5.73	1.44	6.29	1.00	7.00	4.96	6.89
Knowledge	172	4.13	1.51	4.00	1.00	7.00	3.25	5.00
GAP								
Beliefs	172	56.33	14.17	57.0	15	75	51	67.00
Behaviors	172	48.56	14.45	48.5	15	75	39	58.25

### 3.3 Factors associated with gay-affirmative beliefs

In univariable analyses, higher LGBT-DOCSS scores, older age, longer work experience, previous LGBT-related training, experience working with LGBT patients, having close relationships with LGBT individuals, and left-wing political orientation were associated with higher Beliefs scores. In contrast, male sex, higher monthly working hours, religiosity, and right-wing political orientation were associated with lower Beliefs scores. Detailed results of the univariable analyses are presented in Table 3.

**Table 3:** Univariable and multivariable linear regression analyses of factors associated with gay-affirmative beliefs (GAP beliefs domain).

Predictor		Univariable analysis				Multivariable analysis			
		*β*	95% CI		*p*	*β*	95% CI		*p*
Total LGBT-DOCSS score		8.263	6.128	10.397	<0.001 *	3.853	1.211	6.495	0.005 *
Clinical preparedness		−0.436	−2.169	1.297	0.62				
Attitudes		6.043	4.871	7.215	<0.001 *				
Knowledge		3.112	1.774	4.45	<0.001 *				
Gender	Female	Ref.				Ref.			
	Male	−7.281	−11.433	−3.13	0.001 *	−2.672	−6.311	0.967	0.149
	Other	−14.932	−42.218	12.354	0.282	−3.306	−35.364	28.752	0.839
Age [years]		0.468	0.108	0.827	0.011 *	−0.114	−0.686	0.457	0.694
Place of residence	Rural area	Ref.							
	City up to 20 th. inhab.	−0.283	−11.853	11.288	0.962				
	City 20–100 th. inhab.	0.282	−7.476	8.039	0.943				
	City 100–500 th. inhab.	1.124	−6.582	8.829	0.774				
	City over 500 th. inhab.	2.871	−3.817	9.56	0.398				
Principal place of work	Emergency Medical Team	Ref.							
	Emergency Department	3.031	−1.891	7.953	0.226				
	Hospital ward other than the ED	4.315	−3.913	12.543	0.302				
	Other	7.202	−0.528	14.932	0.068				
Sexual Orientation	Heterosexual	Ref.				Ref.			
	Homosexual	10.246	3.951	16.541	0.002 *	−0.923	−6.875	5.029	0.76
	Bisexual	10.511	4.456	16.567	0.001 *	1.029	−4.518	6.576	0.714
	I’d rather not to tell/other	−2.706	−21.74	16.327	0.779	−9.269	−31.634	13.095	0.414
Marital status	Single, divorced	Ref.							
	Married or in formalized partnership	0.808	−4.899	6.515	0.78				
	Informal relationship	1.57	−3.624	6.764	0.552				
Education	High school or bachelor’s degree	Ref.							
	Master’s degree or higher	−1.803	−6.594	2.988	0.459				
Work experience [years]		0.556	0.109	1.003	0.015 *	0.561	−0.116	1.239	0.104
Average number of working hours per month [h]		−0.056	−0.093	−0.018	0.004 *	−0.013	−0.044	0.018	0.413
Has the topic of LGBT health been addressed in your previous education (studies/post-secondary education)?	No,	Ref.				Ref.			
	Yes	−2.697	−8.605	3.212	0.369	−2.198	−6.965	2.57	0.364
	I do not remember	−11.684	−21.188	−2.18	0.016 *	−6.024	−13.813	1.765	0.129
LGBT-related trainings (conferences, webinars) in last 5 years	No	Ref.				Ref.			
	Yes	13.057	2.416	23.698	0.016 *	0.415	−8.746	9.576	0.929
Experience working with LGBT patients outside of formal training	No	Ref.				Ref.			
	Yes	6.095	1.281	10.909	0.013 *	0.486	−3.57	4.541	0.813
Do you feel prepared to work with LGBT patients in terms of content and practice?	No	Ref.							
	Yes	−2.721	−7.643	2.201	0.277				
Religious beliefs	Non-religious	Ref.				Ref.			
	Religious	−5.843	−10.051	−1.634	0.007 *	0.746	−3.022	4.513	0.696
Close relationships with LGBT persons (friends, family)	No	Ref.				Ref.			
	Yes	13.661	9.088	18.234	<0.001 *	6.403	1.97	10.837	0.005 *
Overall political views	Centrist	Ref.				Ref.			
	Left-wing	8.895	4.1	13.689	<0.001 *	3.742	−1.358	8.842	0.149
	Right-wing	−15.284	−20.656	−9.913	<0.001 *	−12.256	−17.527	−6.985	<0.001 *
	I cannot assess	−1.64	−6.405	3.126	0.498	−1.428	−5.876	3.019	0.527
Do you think your personal beliefs can influence the way you work with LGBT people?	No	Ref.				Ref.			
	Yes	5.83	1.635	10.026	0.007 *	0.363	−3.232	3.957	0.842

In the multivariable model, higher overall LGBT-DOCSS scores (*β* = 3.85, 95% CI: 1.21–6.50, *p* = 0.005) and having close relationships with LGBT individuals (*β* = 6.40, 95% CI: 1.97–10.84, *p* = 0.005) were independently associated with higher gay-affirmative beliefs. Conversely, right-wing political orientation was independently associated with lower Beliefs scores (*β* = −12.26, 95% CI: −17.53 to −6.99, *p* < 0.001). Detailed results of the multivariable analysis are presented in Table 3.

### 3.4 Factors associated with gay-affirmative behaviors

In univariable analyses, higher LGBT-DOCSS scores, older age, longer work experience, participation in LGBT-related training, previous experience working with LGBT patients, having close relationships with LGBT individuals, and left-wing political orientation were associated with higher Behaviors scores. Conversely, male sex, higher monthly working hours, religiosity, and right-wing political orientation were associated with lower Behaviors scores. Detailed results of the univariable analyses are presented in Table 4. In the multivariable model, higher overall LGBT-DOCSS scores were independently associated with higher gay-affirmative behaviors (*β* = 7.29, 95% CI: 4.62–9.96, *p* < 0.001). In contrast, right-wing political orientation was independently associated with lower Behaviors scores (*β* = −6.74, 95% CI: −12.07 to −1.41, *p* = 0.013). Detailed results of the multivariable analysis are presented in Table 4.

**Table 4:** Univariable and multivariable linear regression analyses of factors associated with gay-affirmative behaviors (GAP Behaviors domain).

Predictor		Univariable analysis				Multivariable analysis			
		*β*	95% CI		*p*	*β*	95% CI		*p*
Total LGBT-DOCSS score		10.361	8.385	12.337	<0.001 *	7.293	4.623	9.963	<0.001 *
Clinical preparedness		1.492	−0.263	3.246	0.095				
Attitudes		6.055	4.847	7.263	<0.001 *				
Knowledge		3.942	2.624	5.26	<0.001 *				
Gender	Female	Ref.				Ref.			
	Male	−7.212	−11.462	−2.962	0.001 *	−3.045	−6.723	0.633	0.104
	Other	−7.08	−35.011	20.852	0.617	−1.287	−33.686	31.112	0.938
Age [years]		0.554	0.19	0.918	0.003 *	0.052	−0.525	0.63	0.858
Place of residence	Rural area	Ref.							
	City up to 20 th. inhab.	2.283	−9.526	14.092	0.703				
	City 20–100 th. inhab.	1.396	−6.522	9.313	0.728				
	City 100–500 th. inhab.	3.439	−4.426	11.304	0.389				
	City over 500 th. inhab.	2.962	−3.864	9.788	0.393				
Principal place of work	Emergency Medical Team	Ref.							
	Emergency Department	2.802	−2.22	7.824	0.272				
	Hospital ward other than the ED	5.373	−3.024	13.769	0.208				
	Other	6.752	−1.136	14.64	0.093				
Sexual Orientation	Heterosexual	Ref.				Ref.			
	Homosexual	13.159	6.783	19.534	<0.001 *	2.005	−4.01	8.02	0.511
	Bisexual	9.084	2.951	15.217	0.004 *	0.23	−5.376	5.836	0.935
	I’d rather not to tell/other	3.302	−15.976	22.579	0.736	−8.427	−31.029	14.175	0.463
Marital status	Single, divorced	Ref.							
	Married or in formalized partnership	2.312	−3.494	8.119	0.433				
	Informal relationship	2.779	−2.505	8.064	0.301				
Education	High school or bachelor’s degree	Ref.							
	Master’s degree or higher	−2.203	−7.085	2.68	0.374				
Work experience [years]		0.584	0.128	1.039	0.012 *	0.336	−0.349	1.021	0.333
Average number of working hours per month [h]		−0.06	−0.098	−0.021	0.003 *	−0.018	−0.05	0.014	0.26
Has the topic of LGBT health been addressed in your previous education (studies/post-secondary education)?	No,	Ref.				Ref.			
	Yes	−2.564	−8.604	3.476	0.403	−1.531	−6.349	3.287	0.531
	I do not remember	−11.081	−20.797	−1.365	0.026 *	−5.826	−13.698	2.046	0.146
LGBT-related trainings (conferences, webinars) in last 5 years	No	Ref.				Ref.			
	Yes	14.906	4.102	25.709	0.007 *	−1.162	−10.421	8.096	0.804
Experience working with LGBT patients outside of formal training	No	Ref.				Ref.			
	Yes	9.118	4.314	13.922	<0.001 *	2.524	−1.575	6.623	0.226
Do you feel prepared to work with LGBT patients in terms of content and practice?	No	Ref.							
	Yes	3.039	−1.976	8.054	0.233				
Religious beliefs	Non-religious	Ref.				Ref.			
	Religious	−6.42	−10.696	−2.143	0.003 *	0.436	−3.371	4.244	0.821
Close relationships with LGBT persons (friends, family)	No	Ref.				Ref.			
	Yes	11.525	6.713	16.336	<0.001 *	1.148	−3.332	5.629	0.613
Overall political views	Centrist	Ref.				Ref.			
	Left-wing	10.353	5.224	15.482	<0.001 *	3.229	−1.925	8.384	0.218
	Right-wing	−10.916	−16.662	−5.17	<0.001 *	−6.741	−12.068	−1.413	0.013 *
	I cannot assess	−1.794	−6.892	3.304	0.488	−0.917	−5.412	3.578	0.688
Do you think your personal beliefs can influence the way you work with LGBT people?	No	Ref.				Ref.			
	Yes	4.892	0.583	9.201	0.026 *	−0.722	−4.355	2.911	0.695

*Statistically significant (*p* < 0.05).

## 4 Discussion

The present study addressed an important gap in the literature by examining factors associated with gay-affirmative beliefs and behaviors among Polish paramedics. Consistent with the study objective, LGBT clinical competence was independently associated with both affirmative beliefs and behaviors, while close relationships with LGBT individuals and political orientation were associated with selected dimensions of gay-affirmative practice.

LGBT clinical competence was independently associated with both affirmative beliefs (*β* = 3.85) and behaviors, with a notably stronger association for behaviors (*β* = 7.29). Notably, LGBT clinical competence demonstrated one of the strongest independent associations observed in this study, underscoring its potential relevance as a target for intervention. This pattern is consistent with social-cognitive and planned-behavior models of affirmative practice, in which knowledge, attitudes, and self-efficacy translate into affirmative professional behaviors (27, 28). Alessi and colleagues demonstrated that training and affirmative attitudes operate largely through affirmative self-efficacy and beliefs to shape engagement in affirmative practice (28), while attitudes toward affirmative practice have been identified as the single strongest predictor of the intention to provide such care (27).

Our findings are also broadly consistent with previous studies using the LGBT-DOCSS instrument. Among nurses, physicians, and allied health professionals, LGBT-focused education and training have been associated with higher levels of LGBT-related knowledge, greater clinical preparedness and self-perceived competence, and more inclusive healthcare practices (16, 29–33). The present study extends this evidence to paramedics, who represent a frontline professional group not previously examined in relation to gay-affirmative practice, and suggests that the clinical preparedness underlying competence may be particularly consequential for what practitioners actually do at the bedside, beyond what they believe.

The prehospital context lends this finding particular weight. Unlike many other healthcare settings, prehospital encounters are brief, occur under substantial time pressure, and often require rapid communication and trust-building with a patient met only moments earlier (34, 35). In such circumstances, deficits in LGBT-related competence may be especially consequential because clinicians have limited opportunity to compensate through prolonged interaction or to repair an early misstep over the course of an ongoing therapeutic relationship. This interpretation is reinforced by our earlier national study, in which Polish paramedics reported relatively favorable attitudes toward LGBT patients but markedly limited clinical preparedness (17). Taken together, the two studies suggest that the principal deficit in this workforce lies not in willingness but in preparedness and that competence may therefore represent a potentially modifiable factor associated with more affirmative practice. Importantly, competence is a modifiable attribute: prior evidence indicates that even brief, skills-based LGBT training can improve clinical preparedness and foster more inclusive practice, supporting a pathway from training to competence to affirmative practice (29, 33).

Having close relationships with LGBT individuals was independently associated with more affirmative beliefs, but not with affirmative behaviors. This dissociation is theoretically meaningful and aligns with intergroup contact theory, which holds that personal contact with members of a stigmatized group reduces prejudice and fosters more favorable attitudes (36). A large meta-analysis confirmed that intergroup contact typically reduces prejudice across a wide range of outgroups, including sexual minorities, and that intergroup friendship is an especially powerful component of this effect (36). That close relationships were associated with beliefs but not behaviors are consistent with the proposition that personal contact primarily reshapes the attitudinal and affective dimensions of affirmation, whereas professional conduct, particularly under the time-critical conditions of prehospital care, may depend more heavily on formal preparedness than on private attitudes. This again points to clinical competence, rather than personal exposure alone, as the more actionable target for ensuring consistent affirmative behavior at the point of care. Although personal contact with LGBT individuals cannot be directly modified through educational interventions, training programs may partially address this gap by incorporating educators with lived experience, perspective-taking exercises, and implicit bias training. Such approaches may help reduce stereotyping and foster more affirming attitudes toward sexual minority patients, particularly when supported by inclusive institutional environments and organizational norms (37, 38).

A particularly salient finding was that right-wing political orientation was independently and consistently associated with lower affirmative beliefs (*β* = −12.26) and behaviors (*β* = −6.74), representing one of the strongest associations observed in our models. This is consistent with a broad body of evidence indicating that political conservatism is consistently associated with prejudice toward sexual minorities, including in healthcare settings, and with constructs such as right-wing authoritarianism and social dominance orientation, which have been linked to less favorable attitudes toward LGBT individuals across cultural contexts (39, 40). It is also consistent with our earlier work, in which religiosity, which in some populations is associated with more conservative political attitudes was related to lower attitudinal awareness and basic knowledge in this group (17).

This finding must be interpreted with considerable caution. First, the cross-sectional design precludes any causal interpretation. The data describe a statistical association, not evidence that political orientation causes less affirmative care, nor do they capture the complex pathways: socialization, values, exposure, and education, that may underlie such an association. Political orientation is best interpreted as a marker of a broader constellation of attitudes, values, and social experiences rather than as an explanatory mechanism. Second, this association may be partially shaped by selection processes: in our previous national study, incomplete-response patterns suggested that participants with right-leaning views or strong religious convictions were more likely to discontinue the survey early (17). Given the shared recruitment approach, a similar dynamic cannot be excluded here, which may have influenced the magnitude and precision of this estimate. Third, our intent is strictly descriptive and scientific: we do not evaluate any political position, nor suggest that views are incompatible with professional, equitable care. Rather, the central implication is pragmatic, because political orientation is neither an appropriate nor a feasible target for intervention, efforts aimed at strengthening gay-affirmative practice may focus on modifiable factors, most notably LGBT clinical competence. Educational interventions may be especially valuable because they can operate independently of personal worldview, framing inclusive care as a matter of professional standards and patient safety rather than ideology (33).

Collectively, these findings carry a coherent practical implication. Of the factors independently associated with gay-affirmative practice in this study, clinical competence is the most readily modifiable through education and professional development, whereas political orientation and personal relationships are largely not. Notably, only 15.1% of participants reported that LGBT health had been addressed in their formal education, and just 4.1% had attended relevant training in the preceding 5 years, indicating a substantial and addressable gap. Embedding structured, skills-based LGBT health content into paramedic curricula and continuing professional development, an approach also endorsed by professional emergency-medicine bodies (23) may therefore represent one potential approach to strengthening equitable care for gay, lesbian, bisexual, and transgender patients in prehospital settings. Beyond undergraduate and continuing education, healthcare organizations may also benefit from incorporating LGBT-inclusive competencies into professional standards, clinical guidelines, and quality-improvement initiatives. Taken together, these findings suggest that LGBT clinical competence represents a potentially important and modifiable factor associated with gay-affirmative practice.

### 4.1 Study limitations

Several limitations should be considered when interpreting the findings of this study. First, the cross-sectional design precludes any conclusions regarding causality. Therefore, the identified associations between LGBT clinical competence and gay-affirmative beliefs and behaviors should not be interpreted as causal relationships. Second, participants were recruited using a convenience sampling strategy through social media and professional networks, which may have introduced selection bias and limited the representativeness of the sample. Individuals with greater interest in LGBT-related issues may have been more likely to participate. Third, the study relied on self-reported measures, making the results potentially susceptible to recall bias and social desirability bias. Participants may have overestimated their LGBT clinical competence or the extent to which they engage in affirmative professional behaviors. Additionally, because sexual orientation was self-reported, some participants may have chosen not to disclose their identity due to privacy concerns or perceived stigma, potentially resulting in an underrepresentation of sexual minority participants in the study sample. Furthermore, although the GAP assesses affirmative beliefs and self-reported professional behaviors, it does not directly measure actual clinical behavior. Therefore, the observed associations should not be interpreted as direct evidence of actual clinical behavior during real-world clinical encounters. Fourth, although the final sample size was sufficient to conduct the planned analyses, the number of participants included in the final dataset was relatively modest (14). This limitation is not unique to the present study, as recruitment of healthcare professionals for research focused on LGBT-related issues remains challenging in many settings. Nevertheless, the sample size may have reduced statistical power for detecting smaller associations and limited the precision of some estimates. Additionally, a substantial proportion of respondents did not complete the study. Attrition analyses identified significant differences between participants included in the final analytical sample and those who discontinued participation; therefore, attrition bias cannot be excluded and may have influenced the observed associations. Finally, the study was conducted exclusively among paramedics practicing in Poland. Consequently, the findings may not be directly generalizable to other healthcare professions, healthcare systems, or cultural contexts.

## 5 Conclusion

In this study of Polish paramedics, higher LGBT clinical competence was independently associated with more affirmative beliefs and behaviors toward gay and lesbian patients. Additionally, having close relationships with LGBT individuals was associated with more affirmative beliefs, whereas right-wing political orientation was consistently associated with less affirmative beliefs and behaviors. These findings highlight LGBT clinical competence as a factor associated with more affirmative beliefs and behaviors toward gay and lesbian patients. Future research should determine whether educational interventions designed to strengthen LGBT clinical competence translate into more affirmative professional practice among paramedics.

## Data Availability

The raw data supporting the conclusions of this article will be made available by the authors, without undue reservation.

## References

[1] Zeeman L Sherriff N Browne K McGlynn N Mirandola M Gios L . A review of lesbian, gay, bisexual, trans and intersex (LGBTI) health and healthcare inequalities. Eur J Pub Health. (2019) 29:974–80. doi: 10.1093/eurpub/cky226, 30380045 PMC6761838

[2] Medina-Martínez J Saus-Ortega C Sánchez-Lorente MM Sosa-Palanca EM García-Martínez P Mármol-López MI. Health inequities in LGBT people and nursing interventions to reduce them: a systematic review. Int J Environ Res Public Health. (2021) 18:11801. doi: 10.3390/ijerph182211801, 34831556 PMC8624572

[3] Carter EJR Sagaribay R Singh A Evangelista LS Kuhls DA Pharr JR . Bias at the bedside: a comprehensive review of racial, sexual, and gender minority experiences and provider attitudes in healthcare. Healthcare. (2026) 14:114. doi: 10.3390/healthcare14010114, 41517045 PMC12786212

[4] Czapla M Stańczykiewicz B Kurpas D Uchmanowicz I Uchmanowicz B Juárez-Vela R . Polish validation of the LGBTQ+ healthcare experiences scale (LGBTQ+ HCES). Adv Clin Exp Med. (2025) 34:1565–74. doi: 10.17219/acem/209762, 40948467

[5] Sileo KM Baldwin A Huynh TA Olfers A Woo J Greene SL . Assessing LGBTQ+ stigma among healthcare professionals: an application of the health stigma and discrimination framework in a qualitative, community-based participatory research study. J Health Psychol. (2022) 27:2181–96. doi: 10.1177/13591053211027652, 35924592 PMC9642061

[6] Casanova-Perez R Apodaca C Bascom E Mohanraj D Lane C Vidyarthi D . Broken down by bias: healthcare biases experienced by BIPOC and LGBTQ+ patients. AMIA Annu Symp Proc AMIA Symp. (2021) 2021:275–84. 35308990 PMC8861755

[7] Berkman C Stein GL Rosa WE Acquaviva KD Godfrey D Woody I . Discriminatory health care reported by seriously ill LGBTQ+ persons and partners: project respect. Palliat Support Care. (2025) 23:e101. doi: 10.1017/S1478951524001913, 40275808 PMC12145487

[8] Dahlhamer JM Galinsky AM Joestl SS Ward BW. Barriers to health care among adults identifying as sexual minorities: a US National Study. Am J Public Health. (2016) 106:1116–22. doi: 10.2105/AJPH.2016.303049, 26985623 PMC4880242

[9] Quinn GP Sanchez JA Sutton SK Vadaparampil ST Nguyen GT Green BL . Cancer and lesbian, gay, bisexual, transgender/transsexual, and queer/questioning (LGBTQ) populations. CA Cancer J Clin. (2015) 65:384–400. doi: 10.3322/caac.21288, 26186412 PMC4609168

[10] Scott SB Knopp K Yang JP Do QA Gaska KA. Sexual minority women, health care discrimination, and poor health outcomes: a mediation model through delayed care. LGBT Health. (2023) 10:202–10. doi: 10.1089/lgbt.2021.0414, 36521166

[11] Hascher K Jaiswal J LoSchiavo C Ezell J Duffalo D Greene RE . Lack of informed and affirming healthcare for sexual minority men: a call for patient-centered care. J Gen Intern Med. (2024) 39:2023–32. doi: 10.1007/s11606-024-08635-8, 38308157 PMC11306825

[12] Mehmet. *Poland Anti-LGBTI Hate Timeline ILGA-Europe*. (2021). Available online at: https://www.ilga-europe.org/report/poland-anti-lgbti-hate-timeline/ (Accessed June 23, 2026).

[13] Mehmet. *Rainbow Map 2026|ILGA-Europe*. (2026). Available online at: https://www.ilga-europe.org/report/rainbow-map-2026/ (Accessed June 23, 2026).

[14] Karniej P Dissen A Pietrzykowski Ł Juárez-Vela R Sabater AM Kulińska J . Lesbian, gay, bisexual, and transgender clinical competence of health professionals in Poland and Spain: results of the health exclusion research in Europe (HERE) study. BMC Med Educ. (2025) 25:144. doi: 10.1186/s12909-025-06744-4, 39881315 PMC11780758

[15] Karniej P Dissen A Del Pozo-Herce P Juárez-Vela R Martínez-Sabater A Gea-Caballero V . Gay affirmative practices among healthcare professionals in Poland and Spain: results of health exclusion research in Europe (HERE) study. Front Public Health. (2025) 13:1568486. doi: 10.3389/fpubh.2025.1568486, 40331116 PMC12053289

[16] Bidell MP. The lesbian, gay, bisexual, and transgender development of clinical skills scale (LGBT-DOCSS): establishing a new interdisciplinary self-assessment for health providers. J Homosex. (2017) 64:1432–60. doi: 10.1080/00918369.2017.1321389, 28459378

[17] Czapla M Karniej P Juárez-Vela R Kędzierski K Dissen A Griesmann K . Clinical competence of polish paramedics in caring for LGBT patients: a national cross-sectional study. Adv Clin Exp Med. (2026) 35:536. doi: 10.17219/acem/216536, 42012480

[18] Wille L Jewell T Wolfe A Peterson E Shaughnessy A Roblee C . Insufficient LGBTQ+ education across disciplines suggested by national survey of health professionals in training. PLoS One. (2025) 20:e0316931. doi: 10.1371/journal.pone.0316931, 39761251 PMC11703085

[19] Salim SM Ameen S Chenganakkattil S Valiyapurayil J Anilal L. LGBTQIA+ cultural competency of psychiatrists in an Indian state: a cross-sectional study. J Homosex. (2026) 73:181–202. doi: 10.1080/00918369.2025.2469572, 40042251

[20] Nowaskie DZ Dauterman JW Dauterman LC Menez OU. S. Pediatric residents’ preparedness, attitudes, and knowledge in LGBTQ+ health care. J Pediatr Health Care. (2024) 38:140–7. doi: 10.1016/j.pedhc.2023.12.002 38429026

[21] Crisp C. The gay affirmative practice scale (GAP): a new measure for assessing cultural competence with gay and lesbian clients. Soc Work. (2006) 51:115–26. doi: 10.1093/sw/51.2.115, 16858917

[22] Zhang H Chen L Fei W Chen S Daihong JI. Translating and validating the gay affirmative practice scale for nurses in mainland China. Sex Med. (2024) 12:73. doi: 10.1093/sexmed/qfae073, 39469532 PMC11514059

[23] Hong T Case V Farcas AM Whitfield D Muller G Schlesinger SA . Caring for transgender and gender diverse prehospital patients: a NAEMSP position statement and resource document. Prehosp Emerg Care. (2025) 29:302–14. doi: 10.1080/10903127.2024.2411723, 39422378

[24] Jalali S Levy MJ Tang N. Prehospital emergency care training practices regarding lesbian, gay, bisexual, and transgender patients in Maryland (USA). Prehospital Disaster Med. (2015) 30:163–6. doi: 10.1017/S1049023X15000151, 25723881

[25] Karniej P Dissen A Juarez-Vela R Gea-Caballero V Echániz-Serrano E Czapla M. Psychometric properties and cultural adaptation of the polish version of the lesbian, gay, bisexual, and transgender development of clinical skills scale (LGBT- DOCSS-PL). J Homosex. (2025) 72:45–59. doi: 10.1080/00918369.2024.2302970, 38266174

[26] Karniej P Dissen A Juárez-Vela R Santolalla-Arnedo I Sufrate-Sorzano T Garrote-Camara ME . Psychometric properties and cultural adaptation of the polish version of the gay affirmative practice scale. Front Public Health. (2024) 12:1384429. doi: 10.3389/fpubh.2024.1384429, 38756887 PMC11097662

[27] Alessi EJ Dillon FR Kim HMS. Determinants of lesbian and gay affirmative practice among heterosexual therapists. Psychotherapy. (2015) 52:298–307. doi: 10.1037/a0038580, 25706059

[28] Warren AR Steffen AM Wayland S. Predicting gay affirmative practice from the theory of planned behavior. J Gerontol Soc Work. (2015) 58:671–83. doi: 10.1080/01634372.2015.1075237, 26317370

[29] Kuney MA Noble MD Stubbs DM. LGBTQIA+ cultural competency in healthcare education programs: a scoping review. Nurse Educ Pract. (2025) 84:104333. doi: 10.1016/j.nepr.2025.104333, 40174474

[30] Elboim-Gabyzon M Klein R. Lesbian, gay, bisexual, and transgender clinical competence of physiotherapy students in Israel. BMC Med Educ. (2024) 24:729. doi: 10.1186/s12909-024-05679-6, 38970017 PMC11227150

[31] Pratt-Chapman ML Phillips S. Health professional student preparedness to care for sexual and gender minorities: efficacy of an elective interprofessional educational intervention. J Interprof Care. (2020) 34:418–21. doi: 10.1080/13561820.2019.1665502, 31544550

[32] Kelleher ST Barrett MJ Durnin S Fitzpatrick P Higgins A Hall D. Staff competence in caring for LGBTQ+ patients in the paediatric emergency department. Arch Dis Child. (2023) 108:525–9. doi: 10.1136/archdischild-2022-325151, 37094883 PMC10314017

[33] Donisi V Amaddeo F Zakrzewska K Farinella F Davis R Gios L . Training healthcare professionals in LGBTI cultural competencies: exploratory findings from the Health4LGBTI pilot project. Patient Educ Couns. (2020) 103:978–87. doi: 10.1016/j.pec.2019.12.007, 31866197

[34] Wibring K Magnusson C Axelsson C Lundgren P Herlitz J Andersson Hagiwara M. Towards definitions of time-sensitive conditions in prehospital care. Scand J Trauma Resusc Emerg Med. (2020) 28:7. doi: 10.1186/s13049-020-0706-3, 31996233 PMC6988345

[35] Khani J Dadashzadeh A Gilani N Rahmani A Tabrizi FJ. Prehospital emergency services: expectations of patients and companions. Int J Emerg Med. (2026) 19:3. doi: 10.1186/s12245-025-01115-8, 41491112 PMC12777377

[36] Pettigrew TF Tropp LR. A meta-analytic test of intergroup contact theory. J Pers Soc Psychol. (2006) 90:751–83. doi: 10.1037/0022-3514.90.5.751, 16737372

[37] Morris M Cooper RL Ramesh A Tabatabai M Arcury TA Shinn M . Training to reduce LGBTQ-related bias among medical, nursing, and dental students and providers: a systematic review. BMC Med Educ. (2019) 19:325. doi: 10.1186/s12909-019-1727-3, 31470837 PMC6716913

[38] Onyeador IN Wittlin NM Burke SE Dovidio JF Perry SP Hardeman RR . The value of interracial contact for reducing anti-black Bias among non-black physicians: a cognitive habits and growth evaluation (CHANGE) study report. Psychol Sci. (2020) 31:18–30. doi: 10.1177/0956797619879139, 31743078 PMC6966250

[39] Nemli SA Abdollahpour Ranjbar H Turan B. Political conservatism and social distancing from people living with HIV among medical students: mediating roles of negative stereotypes and negative intergroup emotions. BMC Med Educ. (2025) 25:220. doi: 10.1186/s12909-025-06768-w, 39934791 PMC11818025

[40] Hsu HY. “I agree with LGBT rights, but…”: authoritarianism and social dominance orientation underlying hypocritical attitudes of Taiwan society. Front Psychol. (2022) 13:1062748. doi: 10.3389/fpsyg.2022.1062748, 36619016 PMC9815163

